# NONO and RALY proteins are required for YB-1 oxaliplatin induced resistance in colon adenocarcinoma cell lines

**DOI:** 10.1186/1476-4598-10-145

**Published:** 2011-11-25

**Authors:** Serges P Tsofack, Chantal Garand, Chris Sereduk, Donald Chow, Meraj Aziz, David Guay, Hongwei H Yin, Michel Lebel

**Affiliations:** 1 Centre de Recherche en Cancérologie de l'Université Laval, Hôpital Hôtel-Dieu de Québec, 9 McMahon St, Québec, G1R 2J6, Canada; 2 Quebec-Clinical Research Organization in Cancer consortium, (Q-CROC), Canada; 3 Clinical Translational Research Division, Translational Genomics Research Institute, 13208 E Shea Blvd, AZ 85259, USA; 4 Feldan Bio Inc., 4975 Rideau, Suite 100, Québec, G2E 5H5, Canada

## Abstract

**Background:**

YB-1 is a multifunctional protein that affects transcription, splicing, and translation. Overexpression of YB-1 in breast cancers causes cisplatin resistance. Recent data have shown that YB-1 is also overexpress in colorectal cancer. In this study, we tested the hypothesis that YB-1 also confers oxaliplatin resistance in colorectal adenocarcinomas.

**Results:**

We show for the first time that transfection of YB-1 cDNA confers oxaliplatin resistance in two colorectal cancer cell lines (SW480 and HT29 cell lines). Furthermore, we identified by mass spectrometry analyses important YB-1 interactors required for such oxaliplatin resistance in these colorectal cancer cell lines. A tagged YB-1 construct was used to identify proteins interacting directly to YB-1 in such cells. We then focused on proteins that are potentially involved in colorectal cancer progression based on the Oncomine microarray database. Genes encoding for these YB-1 interactors were also examined in the public NCBI comparative genomic hybridization database to determine whether these genes are localized to regions of chromosomes rearranged in colorectal cancer tissues. From these analyses, we obtained a list of proteins interacting with YB-1 and potentially involved in oxaliplatin resistance. Oxaliplatin dose response curves of SW480 and HT29 colorectal cancer cell lines transfected with several siRNAs corresponding to each of these YB-1 interactors were obtained to identify proteins significantly affecting oxaliplatin sensitivity upon gene silencing. Only the depletion of either NONO or RALY sensitized both colorectal cancer cell lines to oxaliplatin. Furthermore, depletion of NONO or RALY sensitized otherwise oxaliplatin resistant overexpressing YB-1 SW480 or HT29 cells.

**Conclusion:**

These results suggest knocking down NONO or RALY significant counteracts oxaliplatin resistance in colorectal cancers overexpressing the YB-1 protein.

## Background

Colorectal cancer is the third leading cause of cancer-related death in the Western world (National Cancer Institute; 2009). Over 50% of those diagnosed require systemic therapy at some point during the disease trajectory. Clinical resistance is almost inevitable for advanced colorectal cancer within 6-12 months of any given therapy. For example, clinical responses of metastatic cancers (*e.g*. death and elimination of cancer cells) to the most advanced therapeutic agents range from 15 to 40%, indicating intrinsic resistance in a majority of colorectal cancer tissues [1]. In addition, acquired resistance almost inevitably occurs in tumors that initially responded [1]. One of the most effective regimens against colorectal cancers, either in adjuvant or in metastatic settings, is the combination of fluoropirimidine and oxaliplatin. Oxaliplatin is a third generation platinum analogue that kills cells by forming adducts on DNA, the most prevalent of which is intra-strand linkage of two adjacent guanines [2]. Nevertheless, the majority of patients with colon cancer are either intrinsically resistant to this drug or become resistant during therapy. Oxaliplatin-resistant cells are characterized by decreased DNA adduct formation [3]. The exact mechanisms responsible for this resistance are still elusive to this date.

Several studies have indicated that the overexpression of YB-1 (Y box-binding protein-1) is related with secondary resistance to cisplatin in melanomas, breast, ovarian, and bladder cancers [4-7]. Furthermore, depletion of YB-1 expression protein with anti-sense RNA against YB-1 specific mRNA results in increased sensitivity to cisplatin [8]. Interestingly, YB-1 is increased in cultured cell lines resistant to cisplatin. In fact, several studies have indicated that the level of nuclear expression of YB-1 is predictive of drug resistance and patient outcome in breast tumors, ovarian cancers, and synovial sarcomas [5,7,9-11]. YB-1 preferentially binds to cisplatin-modified DNA [12]. Further analyzes have indicated that YB-1 actively promotes strand separation of duplex DNA containing either mismatches or cisplatin modifications independently of the nucleotide sequence [13] in addition to having an exonuclease activity [14]. YB-1 was originally described as a transcription regulator that binds to inverted CCAAT box DNA sequences present in the control regions of several genes [15]. In addition to the regulation of transcription, YB-1 is a multifunctional protein that also affects the splicing and the translation of specific mRNAs [16-18]. Several mRNAs regulated by YB-1 are potentially important for chemoresistance [18].

It has been reported that YB-1 expression is increased in colorectal carcinomas compared to normal colon tissues [19]. Although overexpression of YB-1 confers cisplatin resistance in breast and ovarian cancers, it is unknown whether increase YB-1 expression would also confer oxaliplatin resistance in colorectal cancers [20]. In this study, we investigated the impact of YB-1 on oxaliplatin resistance in two different colon adenocarcinoma cell lines. We show that overexpression of YB-1 confers oxaliplatin resistance and a depletion of YB-1 sensitizes cells to oxaliplatin treatments in culture. In addition, we identified by mass spectrometry analyses important YB-1 interactors required for such oxaliplatin resistance in these colorectal cancer cell lines. Knock down analyses of two of these proteins, NONO and RALY, increased oxaliplatin sensitivity in otherwise resistant colorectal cancer cells overexpressing YB-1.

## Methods

### Cell lines and drugs

The human HT29 colon adenocarcinoma cell line was obtained from the American Type Culture Collection (ATCC) and maintained in McCoy's 5A media supplemented with 2 mM L-Glutamine, 10% Fetal Bovine Serum (FBS), and 1% Antibiotic-Antimytotic (Invitrogen, Carlsbad, CA) at 37°C in atmosphere of 5% CO_2_. The human SW480 colon adenocarcinoma cell line (also obtained from ATCC) was maintained in RPMI supplemented with 10% Fetal Bovine Serum (FBS), and 1% Antibiotic-Antimytotic (Invitrogen, Carlsbad, CA) at 37°C in atmosphere of 5% CO_2_. Oxaliplatin was purchased from the Hôtel-Dieu Hospital in Quebec City (Qc, Canada).

### Antibodies

A polyclonal antibody against the N-terminus portion of YB-1 (ab12148) was purchased from Abcam, Inc. (Cambridge, MA). The mouse monoclonal antibody against HNRPB_2_A_1 _(sc-32316) and a rabbit polyclonal antibody against HNRPL (sc-28726) were purchased from Santa Cruz Biotechnology (Santa Cruz, CA). The mouse polyclonal antibody against RALY (H00138046-B030), a mouse polyclonal antibody against MRPL13 (H00028998-B01), and a goat polyclonal antibody against NONO (PAB 7027) were purchased from Abnova Corp. (Taipei City, Taiwan). A rabbit polyclonal antibody against RIBC2 (HPA003210) was purchased from Sigma-Aldrich Inc. (St-Louis, MO). Finally, all horseradish peroxidase-conjugated secondary antibodies (anti-rabbit IgG: NAV934V and anti-mouse IgG: NA931V) were purchased from GE Healthcare Limited (Piscataway, NJ). The above antibodies were used as indicated by the manufacturers. Western blotting analyses were performed as described [13].

### Plasmids and transfections

The YB-1 cDNA was cloned in the pNTAP-B vector in-frame with the TAP-epitope consisting of both calmodulin and streptavidin epitopes (Stratagene, LaJolla, CA). SW480 cells were transfected with Effectene (Qiagen, Inc., Mississauga, ON) and selected in 600 μg/ml of neomycin (Invitrogen, Carlsbad, CA) for two weeks. Colonies were expended and analyzed by Western blotting with an antibody against YB-1 to select TAP-YB-1 expressing clones. The pCMV-YB-1 construct was described before [13]. This YB-1 expression vector was transfected into SW480 cells with the Amaxa nucleofector kit V as described by the manufacturer (Lonza Company, Basel, Switzerland). A transfection efficiency of 19.8% in SW480 cells was routinely obtained with a fluorescent GFP-YB-1 construct using this transfection protocol (data not shown).

### Tandem affinity purification and mass spectrometry analyses

The TAP-YB-1 protein was purified from a stable SW480 clone expressing this protein construct with a TAP purification kit (Stratagene, LaJolla, CA) as described by the manufacturer. We also used large amounts of RNAse A (100 μg/mL) in our extraction buffers to eliminate contaminating ribonucleic acids that could be used as bridging molecules in YB-1 containing complexes. SW480 cells containing an empty TAP vector were used as a control. Eluted proteins were analyzed by SDS-PAGE and lanes corresponding to control TAP and TAP-YB-1 expressing cells were cut into small gel slices. Gel slices were sent to the Proteomics platform of the Quebec Genomic Center (Quebec City, Qc, Canada) for spectrometry analyses and protein identifications. Briefly, gel slices were disposed into 96-well plates and in-gel trypsin digestion was performed on a MassPrep™ liquid handling station (Waters, Mississauga, ON) according to the manufacturer's specifications. Peptide extracts were dried out using a SpeedVac™. Peptide extracts were separated by online reversed-phase nanoscale capillary LC and analyzed by electrospray MS (ES MS/MS). The experiments were performed on a Thermo Surveyor MS pump connected to a LTQ linear ion trap mass spectrometer (Thermo Electron, San Jose, CA) equipped with a nanoelectrospray ion source (Thermo Electron, San Jose, CA). Peptide separation took place within a PicoFrit column BioBasic C18, 10 cm × 0.075 mm internal diameter (New Objective, Woburn, MA) with a linear gradient from 2% to 50% solvent B (acetonitrile, 0.1% formic acid) in 30 min, at 200 nl/min. Mass spectra were acquired using data-dependent acquisition mode (Xcalibure software, version 2.0). Each full-scan mass spectrum (400-2000 m/z) was followed by collision-induced dissociation of the seven most intense ions. The dynamic exclusion function was enabled (30 s exclusion), and the relative collisional fragmentation energy was set to 35%.

All MS/MS samples were analyzed using Mascot (Matrix Science, London, UK; version 2.2.0). Mascot was set up to search against human Uniref_100 protein database assuming a digestion with trypsin. Fragment and parent ion mass tolerance were, respectively, of 0.5 Da and 2.0 Da. Iodoacetamide derivative of cysteine was specified as a fixed modification. Deamidation of asparagine and glutamine, acetylation of lysine and arginine and oxidation of methionine were specified as variable modifications. Scaffold (version 01_07_00; Proteome Software Inc., Portland, OR) was used to validate MS/MS-based peptide and protein identifications. Peptide identifications were accepted if they could be established at > 95.0% probability as specified by the Peptide Prophet algorithm [21]. Protein probabilities were assigned by the Protein Prophet algorithm [22]. Proteins that contained similar peptides and could not be differentiated based on MS/MS analysis alone were grouped to satisfy the principles of parsimony. Using these stringent identification parameters, the rate of false positive identifications is < 1%.

To confirm protein interactions, TAP-YB-1 protein complexes were purified from a stable SW480 clone expressing the TAP-YB-1 construct with a TAP purification kit (Stratagene, LaJolla, CA) as described by the manufacturer. SW480 cells containing an empty TAP vector were used as a control. Eluted proteins were analyzed by SDS-PAGE followed by Western blotting with the appropriate antibodies.

### Oncomine analyses

Oncomine is a cancer microarray database and web-based data-mining platform aimed at facilitating discovery from genome-wide expression analyses. Data can be queried and visualized for a selected gene across microarray analyses available to the public. The parameter for the research in Oncomine included as primary filters the official name of the gene (or protein interacting with YB-1) and the analysis type, which was colorectal cancer *versus *normal tissue analysis. The threshold used to obtain the most significant probes of the queried gene for each microarray data included a two-fold difference in expression between cancers and normal tissues with a *P*-value < 1 × 10^-4^.

### siRNA screening

The antisense technology has been described as a potential anti-cancer therapy for the treatment of several clinical cancers and several molecules are in phase I and II trials [23]. In general, cells were reverse-transfected with a custom arrayed set of 18 genes (4 siRNA sequences/gene) using RNAiMax (Invitrogen, Carlsbad, CA) for SW480 cells or DharmaFect 3 (Dharmacon, Lafayette, CO) for HT29 cells and incubated at 37°C for 24 hours followed by treatment with various concentrations of oxaliplatin and incubated for an additional 96 hours before measuring cell viability via the CellTiter-Glo luminescent assay kit according to the manufacturer's protocol (Promega, Madison, WI). All scramble siRNA controls (GFP, ASNS, and NS) as well as the positive transfection control (Allstar Cell Death Control or ACDC) were purchased from Qiagen (Valencia, CA).

One microliter of 0.667 μM siRNA was printed into bar-coded 384 well solid-white bottom plates (Corning 8749, Lowell, MA) using a Biomek FX Laboratory Automation Workstation for a final assay concentration of 13 nM per well. siRNA buffer [100 mM Potassium Acetate, 30 mM HEPES-KOH, 2 mM Magnesium Acetate (pH 7.6)] was printed into the first two columns of the plate to serve as a control. A transfection reagent solution of RNAiMax and serum free RPMI was added to the plates using a BIO-TEK μFill Microplate Dispenser at 20 μl per well (40 nl RNAiMax/well). The plates containing siRNA and transfection reagents were incubated at room temperature for 30 minutes prior to adding cells to allow complexes to form. Cells were trypsinized, counted, resuspended in 10% FBS assay media, and then seeded at a concentration of 1000 cells/20 μl per well for SW480 cells using the BIO-TEK μFill Microplate Dispenser for a final FBS concentration of 5% (20 μl serum free + 20 μl 10% FBS media). Plates were incubated at 37°C for 24 hours prior to dosing them with 10 μl/well of varying concentrations of oxaliplatin diluted in 5% FBS assay media using a μFill. Initially, twelve different drug concentrations, together with vehicle control, were applied for each control siRNA to obtain a drug dose response curve. Subsequently, six concentrations of oxaliplatin [200, 66.7, 22.2, 7.4, 2.47, 0.82, and 0 μM] spanning the entire response range of the drug were applied to SW480 cells during the siRNA screening process. The first column of every plate (containing siRNA buffer, no siRNA) was left untreated to serve as a control for plate-to-plate variation. Plates were incubated for an additional 96 hours post drug treatment and then read for cell viability. The screen in SW480 cells was performed in two independent runs.

siRNAs altering SW480 sensitivity to oxaliplatin were also tested in HT29 cells. Identical protocols were used for these cells, with the following exceptions: DharmaFECT 3 was delivered at a concentration of 200 nl/well and six concentrations of oxaliplatin [250, 100, 40, 16, 6.4, 2.56, and 0 μM] spanning the entire dose response range of HT29 cells were used for the siRNA screening process based on an initial test with twelve different oxaliplatin concentrations.

The siRNA sequences against YB-1 are 5'-AAGAAGAAAUAUGAAAUUCCA-3' for sequence siRNA-A, 5'-CUGCAAGCACCUGUUAAUAAA-3' for siRNA-B, and 5'-CAGGCGAAGGUUCCCACCUUA-3' for siRNA-C.

### Growth curves and FACS analyses

For growth curves, 10 to 50 thousands cells were plated in 60 mm dishes (or 35 mm dishes when indicated) and counted with a hemacytometer by the trypan blue exclusion technique every other day. For FACS analyses, trypsinized cells were collected and centrifuged on a top bench centrifuge for 2 min. Cell were resuspended in PBS and an equal volume of 95% cold ethanol was added. The next day cells were centrifuged and the pellet was resuspended in a propidium iodide buffer (0.1% citrate; 0.3% NP-40; 0.002 mg/ml of RNAse A; 50 μg/ml propidium iodide at pH 7.4). Cells were incubated 30 min at 37°C and then analyzed on a Coulter^® ^Epics XL-MCL™ Flow Cytometer (Beckman Coulter Canada, Inc., Mississauga, ON). Data were analyzed with the MultiCycle software (Phoenix Flow System, San Diego, CA, USA).

### RT-PCR

The primers used to amplify a 116 base pairs fragment of the HPRT1 (hypoxanthine phosphoribosyltransferase 1) cDNA are HPRTRev 5'-GCACACAGAGGGCTACAATG-3' and HPRTFor 5'-TGAGGATTTGGAAAGGGTGT-3'. The primers used to amplify a 527 base pairs fragment of the YB-1 cDNA are Y159For 5'-CCAGCAAAATTACCAGAAT-3' and UNYB1Rev 5'-TGATGGTAGAGATGGTAAGC-3'. Reverse transcriptase was performed on 300 ng of total RNA with either the HPRTRev or the UNYB1Rev primer. The cDNA was then amplified with Taq DNA polymerase and the appropriate forward primers for each target mRNA. The PCR conditions for the HPRT1 cDNA were 1 min at 94°C, 1 min at 55°C, and 1 min at 72°C for 30 cycles. The PCR conditions for the YB-1 cDNA were 1 min at 94°C, 1 min at 58°C, and 1 min at 72°C for 30 cycles. Amplified products were analyzed on a 2% agarose gel.

## Results

### Overexpression of YB-1 in SW480 colon cancer cells induce oxaliplatin resistance

**Figure 1:**
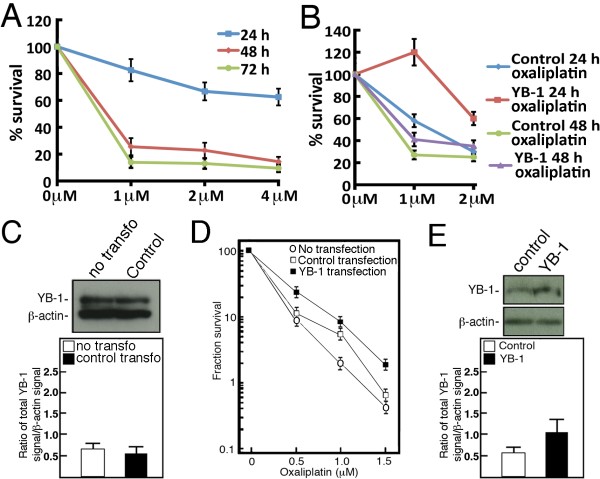
**Impact of YB-1 overexpression on oxaliplatin resistance in SW480 cells**. A) Survival assay of SW480 cells treated for 24, 48, or 72 hours with the indicated concentrations of oxaliplatin. Live cells were then counted with a hemacytometer by the trypan blue exclusion technique. Experiments were performed in duplicate. Bars represent SEM. B) Survival assays of SW480 cells transfected with either wild type YB-1 or an expression empty vector. Six hours after the transfections, cells were treated with one or two μM of oxaliplatin for 24 or 48 hours. Live cells were then counted with a hemacytometer by the trypan blue exclusion technique. Experiments were performed in duplicate. Bars represent SEM. C) Example of Western blots showing endogenous YB-1 expression in control transfected cells (6 hours after transfection) and in nontransfected SW480 cells. The histogram below the Western blots represents the YB-1 signal over β-actin signal. D) Clonogenic assays with SW480 cells transfected either with wild type YB-1 or an empty expression vector. Transfected cells were treated (6 hours after transfection) with the indicated concentrations of oxaliplatin for 18 hours and then plated in 90-mm petri dishes with fresh media without drugs for 10 days. Survival fraction represents the number of colonies after treatment divided by the number of colonies without treatment (in %). Results represent the mean ± SEM of duplicate experiments. E) Example of Western blots showing YB-1 expression in transfected cells (6 hours after transfection). Bands were revealed using an ECL plus kit after less than a five second exposition. The histogram below the Western blots represents the YB-1 signal over β-actin signal.

We first tested the impact of oxaliplatin on SW480 survival. Cells were treated with oxaliplatin for 24, 48 or 72 hours and live cells were counted with trypan blue on a hemacytometer (Figure 1A). SW480 cells were more sensitive after a 48-hour oxaliplatin treatment than only after a 24-hour treatment. We then transfected SW480 colorectal cancer cells with the full-length wild type YB-1 construct or an empty expression vector as a control. Six hours later, transfected cells were treated with oxaliplatin for 24 or 48 hours before counting the cells by the trypan blue exclusion assay with a hemacytometer. As indicated in Figure 1B, YB-1 overexpression induced a two-fold increase in cell survival in the presence of one or two μM oxaliplatin concentrations over the transfected control empty expression vector. The difference between YB-1 and control transfected cells were more evident with a 24-hour oxaliplatin treatment (Figure 1B). We determined whether the transfection protocol had an impact on endogenous YB-1 expression in SW480 cells. YB-1 protein levels in cells transfected with a control empty vector was equivalent to endogenous YB-1 levels in nontransfected cells (Figure 1C). We also performed clonogenic assays to measure overall cell survival after oxaliplatin treatment. Briefly, transfected cells were treated 18 hours with different concentrations of oxaliplatin and then re-plated in fresh media without oxaliplatin for 10 days to allow colony formation. Nontransfected cells were also included as an additional negative control experiment. Clonogenic assays indicated a three-fold difference in the number of colonies at 1.5 μM oxaliplatin between YB-1 overexpressing SW480 cells and transfected or nontransfected control cells (Figure 1D). YB-1 protein levels were on average 1.8-fold higher in YB-1 transfected SW480 cells compared to cells transfected with an empty vector (Figure 1E). All these results indicate that YB-1 overexpression increases oxaliplatin resistance in SW480 cells.

### Identification of YB-1 partners in SW480 colon cancer cells

**Figure 2:**
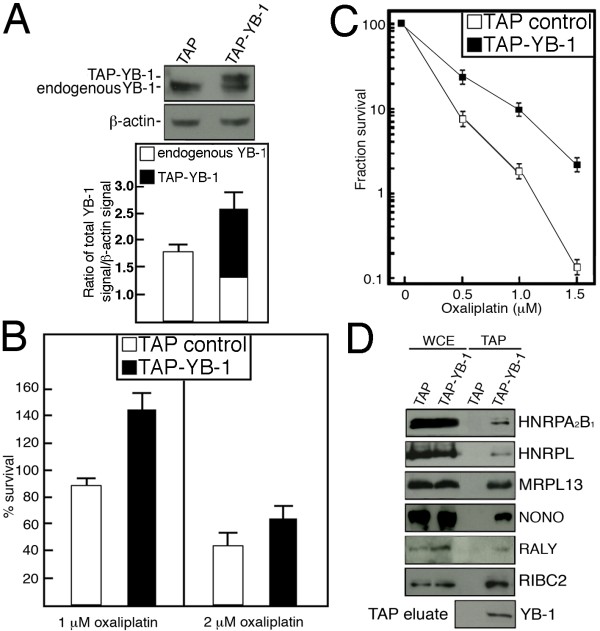
**Increased oxaliplatin resistance in SW480 cells expressing the TAP-YB-1 construct and validation of TAP-YB-1 interaction with six identified protein from the LC-MS/MS**. A) Representative Western blot analysis of SW480 clones expressing the TAP or the TAP-YB-1 constructs. β-actin was used as loading controls. The histogram below the Western blots indicates the endogenous and TAP-YB-1 expression levels in the indicated clones. B) Survival assays of SW480 clones expressing TAP or TAP-YB-1 constructs. Cells were treated with one or two μM of oxaliplatin for 18 h. Live cells were then counted with a hemacytometer by the trypan blue exclusion technique. Experiments were performed in duplicate. Bars represent SEM. C) Clonogenic assays with SW480 clones expressing TAP or TAP-YB-1 constructs. Cells were treated with the indicated concentrations of oxaliplatin for 18 hours and then plated in 90-mm petri dishes with fresh media without drugs for 10 days. Survival fraction represents the number of colonies after treatment divided by the number of colonies without treatment (in %). Results represent the mean ± SEM of duplicate experiments. D) TAP-YB-1 interaction of HNRPA_2_B_1_, HNRPL, MRPL13, NONO, RALY, and RIBC2 with the YB-1 protein. Proteins from TAP and TAP-YB-1 expressing SW480 cells were eluted from the streptavidin beads and analyzed by SDS-PAGE with antibodies against the indicated proteins. Proteins were revealed with an ECL Plus kit. WCE represents the whole cell lysate. TAP represents the protein eluted from the streptavidin beads.

**Figure 3:**
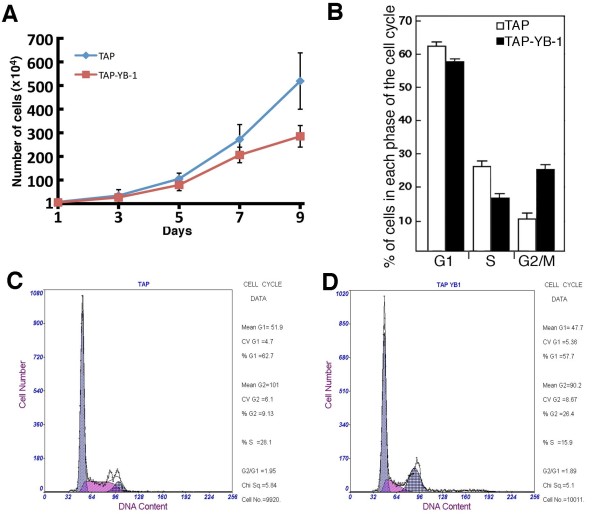
**Cell growth of SW480 cells expressing TAP and TAP-YB-1 constructs**. A) Growth curves of TAP and TAP-YB-1 expressing SW480 clones. B) Graph representing the percentage of the various TAP construct expressing cells in each phase of the cell cycle. C) Example of cell cycle by FACS analyses with SW480 cells expressing a TAP construct. D) Example of cell cycle by FACS analyses with SW480 cells expressing the TAP-YB-1 construct.

The expression of YB-1 in colorectal cancer was queried in the Oncomine public database [24]. Oncomine is a cancer microarray database and web-based data-mining platform aimed at facilitating discovery from genome-wide expression analyses. Data can be queried and visualized for a selected gene across all microarray analyses available to the public. A number of microarray studies have indicated a significant increase in YB-1 expression in colon cancer tissues compared to normal colon tissues [25-27]. In addition, a search of the literature indicated that YB-1 was overexpressed in almost all cancerous lesions in comparison with normal mucosa in surgically resected colorectal carcinomas of 26 patients [19]. Based on such published data, we could hypothesize that YB-1 overexpression may play a role in colorectal cancers. Moreover, it is possible that YB-1 confers oxaliplatin resistance in the context of a protein complex in colon cells. To identify YB-1 protein partners that may also affect oxaliplatin resistance, the YB-1 cDNA was cloned in frame with a TAP tag containing a calmodulin and a streptavidin binding peptide for Tandem Affinity Purification. Such construct was transfected into the SW480 colon cancer cell line. Several stable viable clones were obtained (Figure 2A shows an example of one such clone). The expression level of total YB-1 (endogenous plus TAP-YB-1) was 1.7-fold higher than in SW480 clones expressing the TAP construct alone. The SW480 clones expressing the TAP-YB-1 construct were more resistant to oxaliplatin than cells expressing the TAP control alone (Figure 2B and 2C show an example of one such clone). Thus, stable SW480 clones expressing the TAP-YB-1 behaved like SW480 transiently transfected with a YB-1 expression vector (Figure 1). We next characterized the growth rate of TAP-YB-1 expressing SW480 cells, which are more resistant to oxaliplatin than TAP expressing cells. Figure 3 indicates that TAP-YB-1 expressing SW480 cells had a slower growth rate than TAP expressing SW480 cells (estimated doubling time of 29 and 38 hours for TAP and TAP-YB-1, respectively). TAP-YB-1 expressing SW480 cells tended to accumulate in the G2/M phases of the cell cycle (Figure 3B) compared to TAP expressing cells (Figure 3C and 3D). These results are consistent with previous observation indicating that YB-1 overexpression affects mitosis [28,29].

Co-purification of proteins was achieved on exponentially growing SW480 cells expressing either the TAP alone or the TAP-YB-1 construct. RNAse A was used in the extraction buffer to avoid isolating YB-1 protein complex bound to RNA molecules. Unbound proteins at each step of the chromatography process were removed by extensive washing thus obtaining proteins stringently bound to the TAP-YB-1 construct in cell lysates. Bound proteins were identified by liquid chromatography tandem mass spectrometry (LC MS/MS). The experiment was repeated nine times. Proteins identified from both SW480 TAP and TAP-YB-1 expressing cells were considered artifacts and removed from the final list of potential YB-1 interacting proteins. Additional file 1 gives a list of potential proteins interacting with the TAP-YB-1 construct in SW480 cells. Based on all the identified peptides, there are 128 proteins (excluding YB-1 itself) that potentially can interact with the TAP-YB-1 construct in SW480 cells. Only 67 proteins, however, were identified by more than two peptides in all nine experiments (Additional file 1) with high confidence. We thus continued our analysis on proteins that were identified by at least two peptides in our mass spectrometry data.

We next confirmed the interaction of six of these proteins picked randomly from our list (Additional file 1) by Western blotting analyses (Figure 2D). Total cell lysate from TAP-YB-1 and TAP SW480 clones were incubated with streptavidin binding resin overnight in a cold room and proteins were eluted the next day for immunoblot analyses with antibodies against HNRPA_2_B_1_, HNRPL, MRPL13, NONO, RALY, and RIBC2. As indicated in Figure 2D, all these proteins bound the TAP-YB-1 construct but not the TAP construct alone.

### Identification of YB-1 interactants differentially expressed in colon cancer

To determine whether YB-1 interactants were potentially relevant in colon cancer, we searched for their presence in the Oncomine public database [24]. We queried each YB-1 interacting encoding gene product for their expression levels in colon cancer compared to normal colon tissues. In addition, we determined whether each gene was localized (using the UCSC genome browser at http://genome.ucsc.edu/) to region of the genome rearranged in colon cancer using the comparative genomic hybridization (CGH) database from the NCBI web site http://www.ncbi.nlm.nih.gov/projects/sky/. This database contains 163 colon cancer cases analyzed by the CGH methods (by October 2011). Table 1 indicates the localization of the genes encoding each YB-1 interacting protein and the number of cases, which demonstrated amplification or deletion of the genomic region containing these genes. Overall, 24 genes coding for potential YB-1 interactants were amplified and overexpressed or deleted and underexpressed in colon cancers (Table 1). In addition, based on the literature, five of these genes (RPS7, PABPC1, RPS4X, HNRNPL, and RALY) were up regulated in oxaliplatin resistant ovarian cell lines [30] (Table 1). The other genes did not show significant changes in their expression in colon cancer and were not further considered in this study.

**Table 1:** List of potential YB-1 partners with their chromosomal location and expression levels in colon cancer

			CGH databases (163 colon cancer cases)			ONCOMINE databases	Oxaliplatin
Protein name	Accession number	Chromosome	cases	amplification	deletion	comments	resistance
C1QBP	Q07021	17p13.3	44	8	36	down with grade and recurrence	-
FBRL	P22087	19q13.2	26	25	1	up in tumor vs normal	-
RPS7	P62081	2p25.3	28	26	2	up in tumor vs normal	up
CHCHD9	Q5T1J5	9q21.31	22	19	3	up in tumor vs normal	-
HNRPK	P61978	9q21.32	22	19	3	up in tumor vs normal	-
MRPL19	P49406	2p12	22	22	0	up with grade	-
PABPC1	P11940	8q22.3	62	61	1	up in tumor vs normal	up
MRPL9	Q9BYD2	1q21.3	24	23	1	up in tumor vs normal	-
MRPS7	Q9Y2R9	19p13.12	30	25	5	up with genetic instability	-
PTCD3	Q96EY7	2p11.2	25	25	0	up in tumor vs normal	-
SYNCRIP	O60506	6q14.3	31	26	5	up in tumor vs normal	-
MRPL43	A8K4V4	10q24.31	19	14	5	up with genetic instability	-
SERPINH1	A8K259	11q13.5	28	26	2	up in tumor vs normal	-
RPS4X	P62701	Xq13.1	46	41	5	up in tumor vs normal	up
MRPL44	Q9H9J2	2q36.1	25	23	2	up with genetic instability	-
ILF2	Q12905	1q21.3	24	23	1	up in tumor vs normal	-
NONO	A8K525	Xq13.1	46	41	5	up in tumor vs normal	-
HNRNPL	A6ND69	19q13.2	24	23	1	up with genetic instability	up
LOC138046	O60812	8q21.2	59	58	1	up in tumor vs normal	-
MRPS27	Q92552	5q13.2	23	9	14	up with genetic instability	-
RALY	A8K4T9	20q11.22	92	92	0	up in tumor vs normal	up
MRPL23	A6NGQ5	11p15.5	27	26	1	up in tumor vs normal	-
MBL2	P11226	10q21.1	16	14	2	up in metastasis vs colon primary tumor	-
MRPS14	O60783	1q25.1	25	24	1	up in tumor vs normal	-

### Identification of YB-1 interactants affecting oxaliplatin resistance by the anti-sense technology

**Figure 4:**
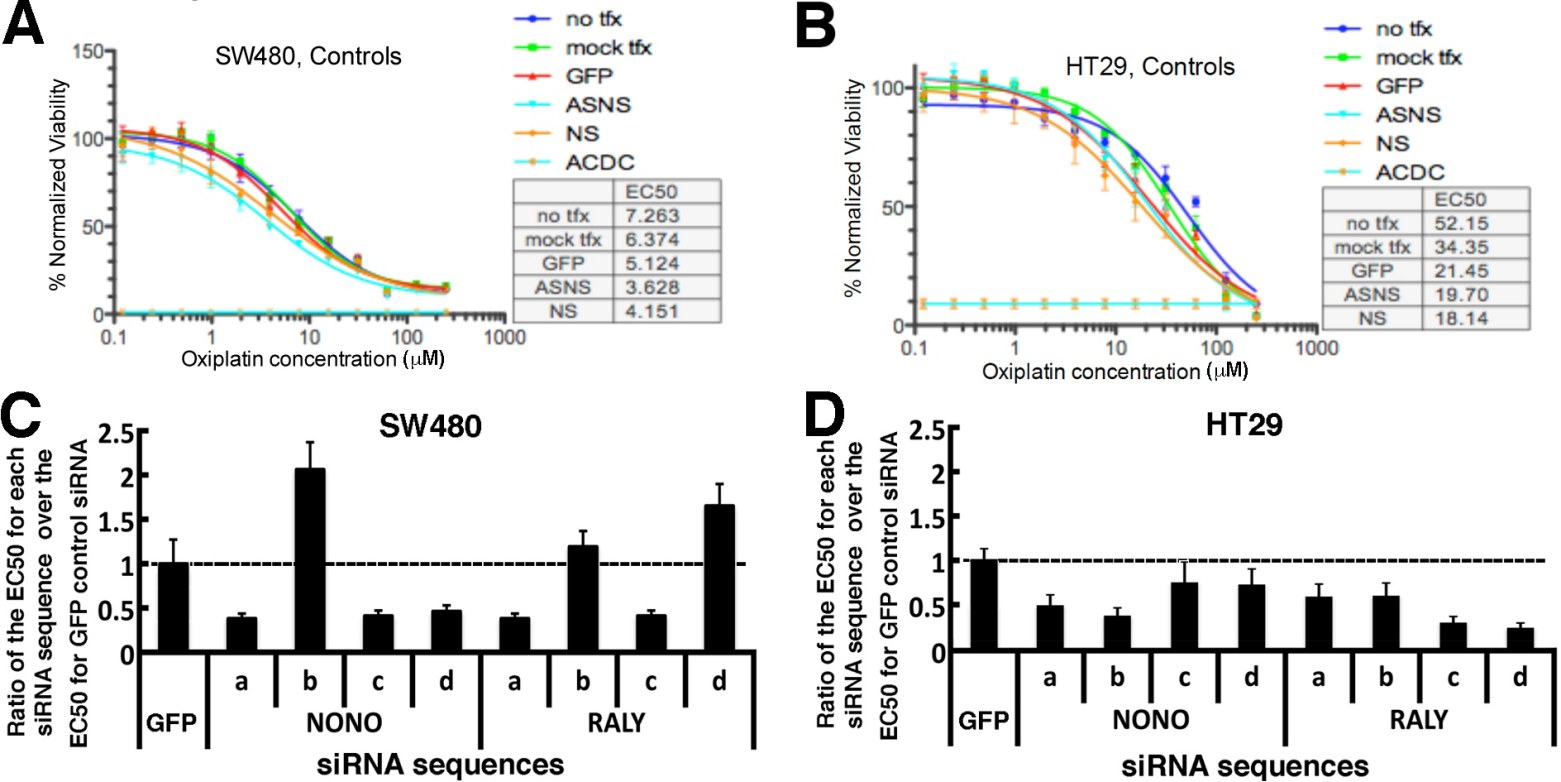
**Graphs showing the changes in oxaliplatin EC50 for each siRNA sequence against controls, NONO, and RALY compared to the oxaliplatin EC50 of cells transfected with a control siRNA against GFP**. A) Oxaliplatin dose response curves obtained with different control siRNAs in SW480 cells. B) Oxaliplatin dose response curves obtained with different control siRNAs in SW480 cells. No tfx indicates no transfection. Mock tfx indicates transfection without a siRNA molecule. NS and All-Star NS (ASNS) are nonspecific siRNA sequences. GFP indicates siRNA against Green Fluorescent Protein. ACDC stands for Allstar Cell Death Control. The ratio (EC50 of the indicated siRNA over the EC50 of transfected siRNA against GFP) is shown for C) SW480 and D) HT29 transfected cells. EC50 values were calculated from the oxaliplatin dose response curves obtained after transfection of the individual siRNA sequence a, b, c, and d targeting the indicated protein. Experiments were performed in duplicate.

To identify YB-1 partners directly involved in oxaliplatin resistance, a high throughput screening with four different small interference RNAs for each target gene (from Table 1) was applied to SW480 cells followed by an analyses in HT29 cells. Two different cell lines were used to determine the reproducibility of our assays in colon adenocarcinoma cells established from different patients. We first evaluated the sensitivity of SW480 and HT29 cells to the drug oxaliplatin by determining their EC50 value. At basal level, SW480 cells were more sensitive to oxaliplatin treatment than HT29 cells. The EC50 value for SW480 and HT29 was 7 μM and 52 μM, respectively (Figure 4A and 4B). We then tested whether the transfection protocols used in this study had any effect on drug sensitivity. We achieved a very high transfection efficiency (> 90% of transfection efficiency) using the RNAiMAX transfection reagents for the SW480 cell line and the DharmaFect 3 transfection reagents for the HT19 cell line (data not shown). Initially, twelve different drug concentrations, together with vehicle control, were applied for each siRNA to obtain a drug dose response curve. As shown in Figure 4A, the transfection of a control siRNA against the Green Fluorescent Protein (GFP) in SW480 did not significantly affect oxaliplatin sensitivity compared to nontransfected SW480 cells. In contrast, the transfection protocol used to obtain > 90% transfection efficiency in HT29 cells significantly increased oxaliplatin sensitivity in this cell line (Figure 4B). Nevertheless, as indicated in Figure 4A and 4B, cells transfected with the different siRNA controls (GFP, NS, ASNS) behaved consistently for both cell lines (EC50 ranging from 3.6-5.1 μM and 18.1-21.4 μM for SW480 and HT29 cells, respectively). We thus used siRNA against GFP as a control reference for our siRNA screening experiments and six different oxalipatin concentrations representing different point along the dose response curves of each cell line as indicated in the materials and methods section and the Additional file 2. Under these conditions, SW480 cells were still more sensitive to oxaliplatin treatments than HT29 cells.

In our first round of screening experiment, we focused our study on genes localized to chromosome amplifications and which are overexpressed in colon cancers based on public databases. Based on the availability of sets of siRNA molecules (*e.g*. four unique siRNA sequences per target), we knocked down 18 targets in SW480 cells and first measure their effects on cell viability (using the CellTiter-Glo luminescent assay) in the absence of oxaliplatin. We eliminated targets that showed cell viability < 50% upon siRNA knockdown in the absence of oxaliplatin (Additional file 3). From this first analysis, there were five sets of siRNAs (representing 27% of the siRNAs screened) demonstrating cytotoxicity > 50% even in the absence of oxaliplatin. We next determined the sensitivity of the remaining 13 targets to oxaliplatin (using siRNA against GFP as a control reference) upon transfection with siRNAs against their corresponding mRNA. To eliminate the risk of off-target effects of a specific siRNA sequence, we prioritized targets that had at least two different siRNA sequences specific to their corresponding mRNA demonstrating the same effect in the presence of oxaliplatin. We identified from this siRNA screen eight proteins of interest that had an impact on cell viability in SW480 cells treated with oxaliplatin upon depletion (data not shown).

**Figure 5:**
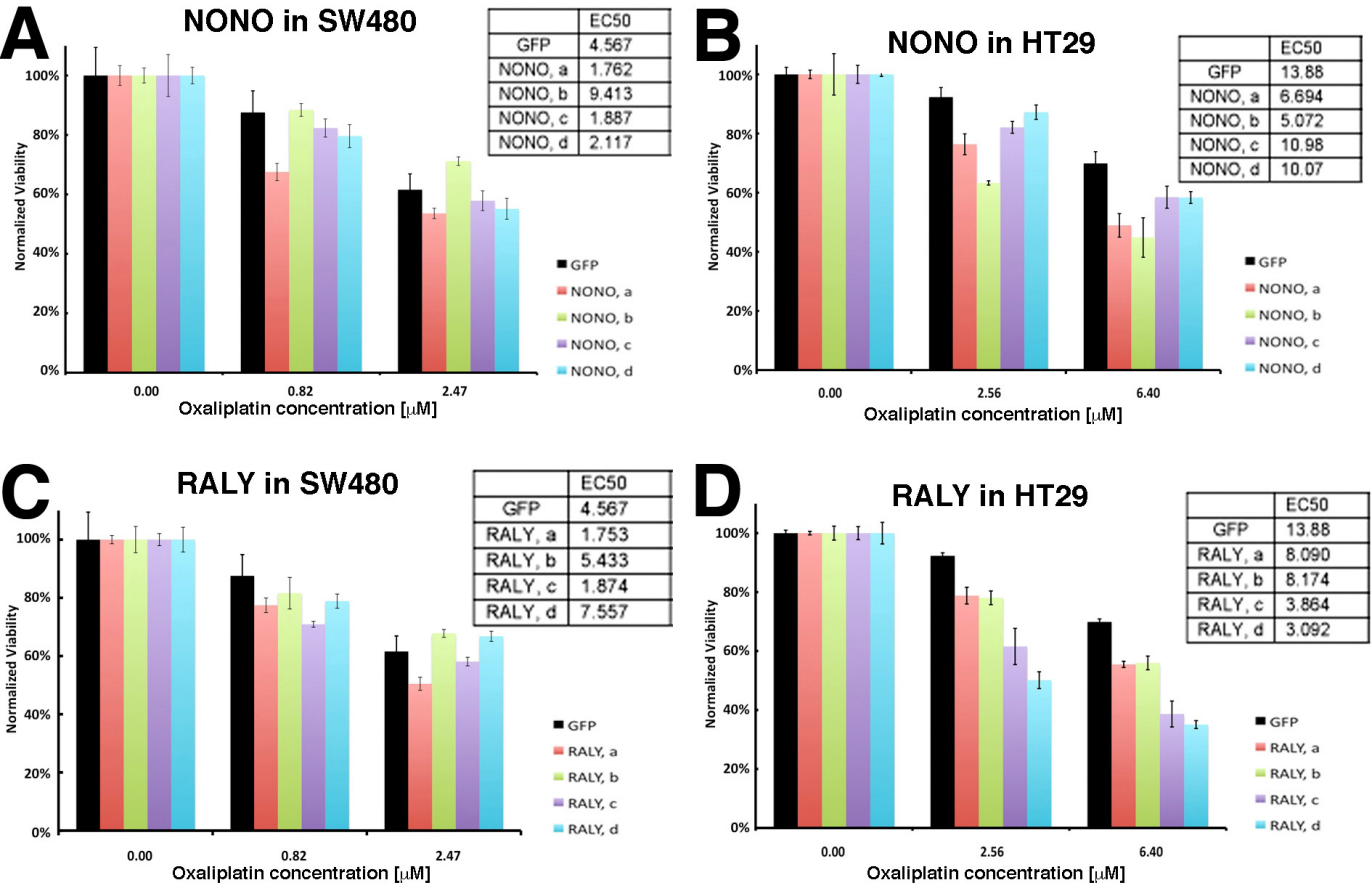
**Histograms showing the response of transfected SW480 and HT29 cells to the drug oxaliplatin**. SW480 cells were transfected with four different siRNA sequences against A) NONO and C) RALY. HT29 cells were transfected with four different siRNA sequences against B) NONO and D) RALY. As controls, cells were transfected with a non-specific siRNA against the green fluorescent protein (GFP). Responses of three oxaliplatin concentrations (out of eight tested) are shown. EC50 for each transfected siRNA is also indicated.

In our second round of siRNA screening experiment, we retested these eight genes (including negative controls) in the presence of various oxaliplatin concentrations from duplicated experiments for both the SW480 and HT29 colon cancer cell lines. The impact of each siRNA on the EC50 value for oxaliplatin compared to control transfected SW480 or HT29 cells is summarized in Table 2. The depletion of only two proteins, NONO and RALY, lead to a greater sensitivity to oxaliplatin in both colon adenocarcinoma cell lines with at least two siRNA sequence molecules. Figure 5 shows examples of normalized response of transfected SW480 and HT29 cells to specific oxaliplatin concentrations and the calculated EC50 values for each siRNA targeting NONO or RALY. Based on the EC50 values, three siRNA molecules against NONO (siRNA a, c, and d) sensitized both SW480 and HT29 cells to the drug oxaliplatin by more than 25% (summarized in Figure 5A and 5B). Two siRNA molecules against RALY (siRNA a and c) sensitized both SW480 and HT29 cells to the drug oxaliplatin by more than 42% (summarized in Figure 5C and 5D). Figure 4C and 4D show the results as the ratio of EC50 for each siRNA sequence over the EC50 value for the control siRNA against GFP. The siRNAs against the other YB-1 interactants that had little effect or opposite effects on oxaliplatin sensitivity in SW480 and HT29 cells were not considered further in this study (effects summarized in Table 2).

**Table 2:** Summary of the impact of siRNAs to deplete eight proteins in SW480 and HT29 colorectal cancer cell lines treated with oxaliplatin (compared to EC50 of siRNA against GFP).

Gene Name	siRNA	Sequence of siRNA	Phenotype in SW480	Phenotype in HT29
FBL	a	CCGGAUGGUCUAGUCUAUGCA	Resistance	Sensitivity
	b	CCUCAUUAACUUGGCCAAGAA	No change	Sensitivity
	c	UUCAUUUGUCGAGGAAAGGAA	Resistance	Sensitivity
	d	CUGCGUAAUGGAGGACACUUU	Resistance	Sensitivity
FLJ20758	a	ACCAUUUAGAUUCAACAUUAA	Resistance	Sensitivity
	b	CAGGACAGAGAGAGUAAAUUA	Resistance	Sensitivity
	c	AACCAGUUAGCUGCUAUAUAA	Resistance	Sensitivity
	d	CCCAGGAAAUAGCCUAUUAAA	No change	No change
HNRPK	a	CAGCAAGUUUAUUAAUCAGAA	Resistance	Sensitivity
	b	CUCUAGUGUCUUAUUAGUUAA	No change	Sensitivity
	c	AUGGAAGUGACUAAUGCUGAA	Resistance	No change
	d	UUCCAUUGUAUGCAAAUUGAA	No change	No change
**NONO**	**a**	**CCAAGUGGACCGCAACAUCAA**	**Sensitivity**	**Sensitivity**
	b	CUGUGUUUGAUUUGUCUCAUA	No change	Sensitivity
	**c**	**AGGCUUGACUAUUGACCUGAA**	**Sensitivity**	**Sensitivity**
	**d**	**AACAAACGUCGCCGAUACUAA**	**Sensitivity**	**Sensitivity**
PABPC1	a	AAACGUAAUUUGGAUUAUAAA	No change	No change
	b	CGGGCUCGGAACACACAUUUA	Resistance	Sensitivity
	c	GACAAAUCCAUUGAUAAUAAA	No change	No change
	d	CCGCACCGUUCCACAGUAUAA	Resistance	Sensitivity
**RALY**	**a**	**CUCAGCCAAGAUCAAGUUAAA**	**Sensitivity**	**Sensitivity**
	b	CGGGCAGACCCUGGACAUCAA	No change	Sensitivity
	**c**	**AAGCAAUGUAACCAACAAGAA**	**Sensitivity**	**Sensitivity**
	d	CACCAUGUCCUUGAAGCUUCA	No change	Sensitivity
RPS7	a	CCGGCUAGUACGCGAAUUGGA	Resistance	Resistance
	b	CACGGGCAAGGAUGUUAAUUU	Resistance	Resistance
	c	CAGCCGGCUCAUAAAGGUUCA	Resistance	Resistance
	d	UUCGAGCGCCAAGAUCGUGAA	Resistance	Resistance
RPS8	a	UGGCUAUGUGCUAGAGGGCAA	Resistance	Resistance
	b	AUCGUGCUCAUCGACAGCACA	Resistance	Resistance
	c	CUCAGAGUGUUGUACUCGUAA	Resistance	Resistance
	d	AAGAGUUGGAGUUCUAUCUUA	Resistance	No change

Genes in bold indicate similar effects with at least two siRNA molecules in both cells lines.

### Validation of NONO and RALY depletions by Western blots

**Figure 6:**
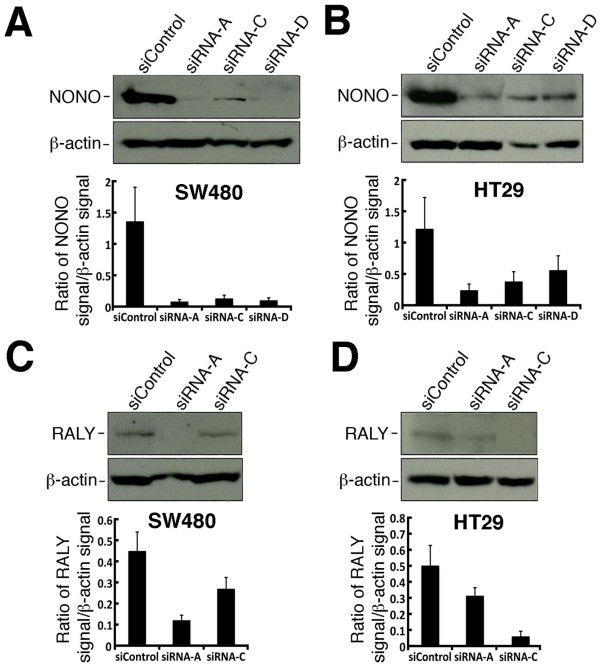
**Knock down of NONO and RALY with specific siRNA molecules in SW480 and HT29 cells**. Cells were transfected with the indicated siRNA molecules and 96 hours later proteins were extracted for Western blot analyses. SW480 and HT29 cells were transfected respectively with siRNAs against NONO and RALY. SiControl represents non-specific siRNA molecules. In every blot, β-actin was used as loading controls. Graph representing densitometric analyses of the Western blots are also shown. Bars in each histogram represent the SEM. Examples of Western blots and scanning analyses of cells transfected with siRNAs against NONO in A) SW480 and B) HT29 cells. Examples of Western blots and scanning analyses of cells transfected with siRNAs against RALY in C) SW480 and in D) HT29 cells.

Based on our results, several siRNAs against NONO and RALY consistently increased oxaliplatin sensitivity in both colon adenocarcinoma (SW480 and HT29) cell lines. We first determined the efficiency of the knock down of these specific siRNA sequences by Western blotting in both cell lines. As shown in Figure 6, siRNA sequence a, c, and d against NONO mRNA significantly decreased NONO protein levels in both SW480 and HT29 cells. These siRNA sequences sensitized SW480 and HT29 cells to oxaliplatin by at least 25% (Figure 5A and 5B) and is consistent with a significant depletion of NONO in the transfected cells (Figure 6A and 6B). Two siRNA sequences against RALY mRNA (siRNA a and c) consistently, decreased RALY protein levels in both SW480 and HT29 cells. These molecules sensitized SW480 and HT29 cells to oxaliplatin (Figure 5C and 5D) and this result is consistent with a significant depletion of RALY in both cell lines (Figure 6C and 6D). The depletion of NONO or RALY protein correlated with oxaliplatin sensitivity in each cell line (Figures 4 and 6).

**Figure 7:**
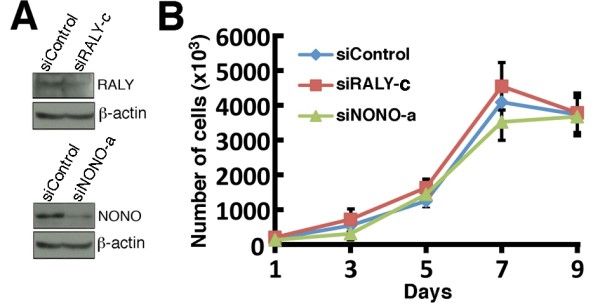
**Growth rate of RALY and NONO depleted cells**. A) Examples of Western blots showing RALY and NONO expression four days after the transfections with the siRNA sequences siRALYc and siNONOa. B) Growth curves of SW480 cells transfected with siRNA sequences against NONO (siNONOa), RALY (siRALYc), or a scrambled control siRNA molecule. Transfected cells were plated in 60 mm petri dishes and live cells were counted every other day with a hemacytometer.

We examined the growth rate of SW480 cells transfected with a scrambled control siRNA or siRNA sequence against NONO (siNONOa) or RALY (siRALYc). As indicated in Figure 7A, the knock down of either RALY or NONO was still efficient four days after the transfection. Nevertheless, the difference in cell growth between transfected cells was not statistically significant (Figure 7B). FACS analyses also indicated no difference in the percentage of cells in each cycle of the cell cycle between siControl, siNONO, and siRALY transfected cells (data not shown). These results indicate that the increased oxaliplatin sensitivity in siNONO or siRALY transfected cells was not due to changes in the rate of cell growth.

### Basal expression of NONO and RALY proteins in SW480 and HT29 cells

**Figure 8:**
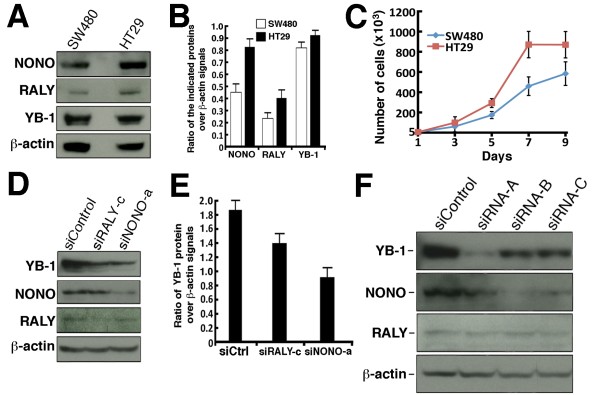
**Co-regulation of NONO and YB-1 in SW480 cells**. A) Basal expression of YB-1, NONO, and RALY in SW480 and HT29 cells. B) Histogram representing the relative expression of YB-1, NONO, and RALY in untransfected cells. Error bars represent the standard error of the mean. C) Growth curves of SW480 and HT29 cells. Cells were plated in 35 mm petri dishes and live cells were counted every other day with a hemacytometer. D) SW480 cells were transfected with siRNA sequences against NONO (siNONOa) or RALY (siRALYc) and 96 hours later cell lysates were extracted for Western analyses with antibodies against NONO, RALY, and YB-1. E) Histogram representing the relative expression of YB-1 in cells transfected with siNONOa or siRALYc. Error bars represent the standard error of the mean. F) SW480 cells were transfected with three different siRNA sequences against YB-1 and 96 hours later cell lysates were extracted for Western analyses with antibodies against NONO, RALY and YB-1. In every blot, β-actin was used as loading controls.

Since a knock down of NONO and RALY increased oxaliplatin sensitivity, we compared the levels of these proteins in the untransfected SW480 and HT29 cells. As mentioned earlier, SW480 cells are more sensitive to oxaliplatin than HT29 cells. Figure 8A and 8B show that the protein levels of NONO and RALY were 70%-80% higher in the more oxaliplatin resistant HT29 cells than the SW480 cells. YB-1 protein levels were 16% higher in HT29 than in SW480 cells. These results indicate a correlation between NONO and RALY expression and oxaliplatin resistance in HT29 and SW480 colorectal cancer cells. Finally, we estimated the growth rate of parental cells in culture (Figure 8C). The estimated doubling time for HT29 and SW480 cells was 29 and 35 hours, respectively.

### Co-regulation of YB-1 and NONO in SW480 cells

We next examined the impact of knocking down NONO and RALY protein levels on YB-1 expression in the parental SW480 cells. Cells were transfected with either a scrambled control siRNA or siRNA sequence against NONO (siNONOa) or RALY (siRALYc) and 96 hours later lysates were extracted for Western blot analyses. As shown in Figure 8D and 8E, siRNA sequence against RALY decreased YB-1 protein levels in SW480 cells by 34%. Knocking down NONO decreased YB-1 protein levels by 50%. Knocking down NONO or RALY with siNONOb or siRALYa molecules also decreased YB-1 protein levels in cells (Additional file 4). In contrast, NONO knock down had no significant effect on RALY expression and RALY knock down had no effect on NONO expression (Figure 8D). Interestingly, a knock down of YB-1 decreased protein levels of NONO protein in SW480 but it had no impact on RALY protein levels in SW480 cells (Figure 8F). These results suggest that YB-1 and NONO regulate each other in SW480 cells.

**Figure 9:**
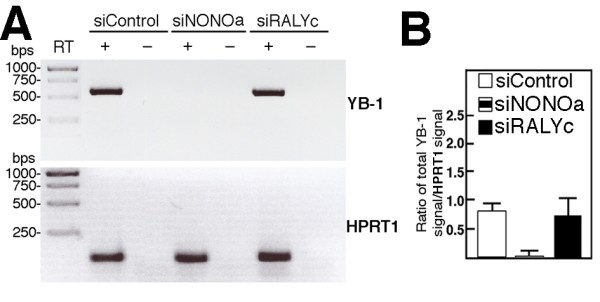
**Impact of depleting endogenous NONO or RALY proteins on YB-1 transcript levels**. A) SW480 cells were transfected with the indicated siRNA molecules and 96 hours later cytoplasmic RNAs were extracted for RT-PCR analyses with primers specific to the YB-1 and HPRT1 mRNAs. All primers used for the PCR step hybridized to two different exons of the target genes. RT indicates the presence or the absence of the reverse transcriptase for the cDNA synthesis step. Experiments were performed in duplicates. B) Graph showing YB-1 mRNA expression relative to the HPRT1 expression in SW480 cells transfected with the indicated siRNA sequences. Bars represent the SEM.

We next examine whether a depletion of NONO or RALY also decreased the mRNA transcript level of YB-1 in addition to its protein level. Cells were transfected with either a scrambled control siRNA or siRNA sequence against NONO (siNONOa) or RALY (siRALYc) and 96 hours later cytoplasmic RNA were isolated for RT-PCR analyses with primers specific for YB-1 and HPRT1 genes. As indicated in Figure 9, a depletion of NONO decreased the mRNA levels of YB-1 but had no effect on the mRNA levels of the control HPRT1 gene. The depletion of RALY had no significant effect on YB-1 and HPRT1 transcript levels compared to the scrambled control siRNA transfection. These results suggest that NONO depletion has an impact on YB-1 expression at the transcriptional stage and a depletion of RALY has an impact on YB-1 expression at the post-transcriptional level.

**Figure 10:**
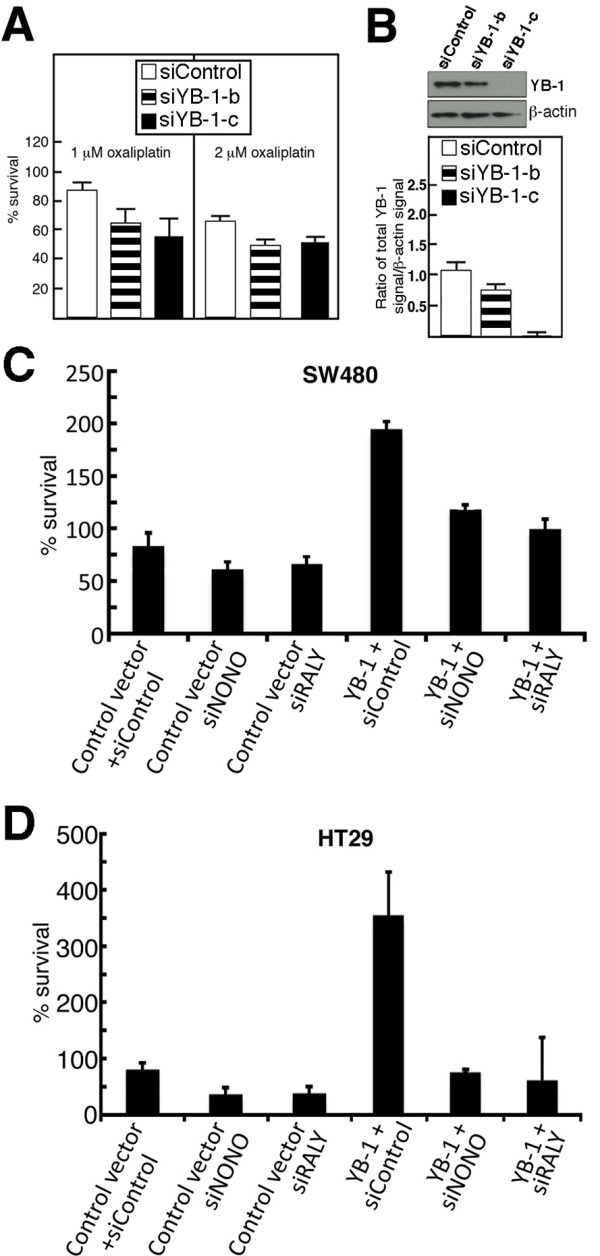
**Impact of depleting endogenous NONO or RALY proteins in YB-1 overexpressing SW480 and HT29 oxaliplatin resistant cells**. A) Survival assays of SW480 cells transfected with either siRNA against YB-1 (siYB-1-b or siYB-1-c) or a nonspecific control siRNA. Three days after the transfections, cells were treated with one or two μM of oxaliplatin for 24 h. Live cells were then counted with a hemacytometer by the trypan blue exclusion technique. Experiments were performed in duplicate. Bars represent SEM. B) Example of Western blots showing YB-1 expression in transfected cells (96 hours after transfection). Bands were revealed using an ECL plus kit after less than five second of exposition. The histogram below the Western blots represents the YB-1 signal over β-actin signal. C) SW480 cells were transfected with the indicated siRNA sequences specific to NONO (siRNA sequence a), RALY (siRNA sequence c), or scrambled control siRNA. Forty-eight hours later, cells were transfected with YB-1 expression vector or empty expression control vector. Transfected cells were plated in 100 mm-petri dishes and were then treated with one μM of oxaliplatin for 16 hours in culture before counting live cells. Results represent the mean ± SEM of quadruplicate experiments. D) HT29 cells were transfected with the indicated siRNA sequences specific to NONO, RALY, or scrambled control siRNA. Forty-eight hours later, cells were transfected with YB-1 expression vector or empty expression control vector. Transfected cells were plated in 100 mm-petri dishes and were then treated with two μM of oxaliplatin for 16 hours in culture before counting live cells. Results represent the mean ± SEM of triplicate experiments.

Finally, we examined the impact of knocking down YB-1 on oxaliplatin sensitivity in parental SW480 cells. As indicated in Figure 10A and 10B, a depletion of YB-1 in SW480 cells with two different siRNA sequences increased oxaliplatin sensitivity.

### The effect of depleting NONO or RALY in YB-1 overexpressing SW480 and HT29 cells

We next examine the impact of knocking down NONO or RALY protein levels in oxaliplatin resistant YB-1 overexpressing SW480 and HT29 cells. Cells were first transfected with the siRNA sequences (siControl, siNONOa, or siRALYc). Three days after this first transfection, cells were re-transfected with an empty vector or a vector expressing YB-1. Cells were treated with two μM of oxaliplatin overnight. The next day cells were counted with a hemacytometer. Knocking down NONO or RALY increased sensitivity of cells to two μM of oxaliplatin by 25-40% (Figure 10C and 10D) as shown previously in Figure 5. Transfection of YB-1 expression vector significantly increased oxaliplatin resistance in SW480 and HT29 cells compared to an empty expression vector by 2.5- and 4.4-fold, respectively. Knocking down the expression of NONO or RALY significantly sensitized YB-1 overexpressing SW480 and HT29 cells to oxaliplatin treatments by 1.5- and 4.4-fold, respectively (Figure 10C and 10D).

### Overexpression of YB-1, NONO, and RALY transcripts in other cancer histotypes

To determine whether YB-1, NONO, and RALY mRNA transcripts could also be increased in other cancer histotypes, we searched the PrognoScan database. PrognoScan is web-based data-mining platform http://gibk21.bio.kyutech.ac.jp/PrognoScan/index.html aimed at facilitating meta-analysis of the prognostic value of genes in different cancers. It provides survival data associated with microarray analyses. The additional file 5 gives a summary of the cancer types in which overexpression of either YB-1, NONO, or RALY are associated with poor outcome of the diseases. To summarize, overexpression of YB-1 was associated with poor prognoses in ovarian, lung, breast, prostate, bladder, head and neck cancers, as well as in multiple myelomas and melanomas (with corrected or COX *P*-values < 0.05). Overexpression of NONO was associated with poor prognoses in lung, brain, breast cancers, and multiple myelomas. Overexpression of RALY was associated with poor survival in ovarian, lung, bladder, brain, and breast cancers as well as in multiple myelomas and melanomas. Interestingly, several studies indicated co-overespression of YB-1 with NONO and/or RALY (highlighted in the table of Additional file 5).

## Discussion

Oxaliplatin has become an effective first-line therapy for colorectal cancer. One major problem with oxaliplatin regimen, however, is the appearance of resistant tumor cells during the course of the treatment. A second problem is the lack of knowledge on a common mechanism that confers oxaliplatin resistance in several colorectal adenocarcinomas. A number of microarray studies have indicated a significant increase in YB-1 expression in colon cancer tissues compared to normal colon tissues [25-27] and one immunohistochemistry study had reached a similar conclusion [19]. In this study, we examined whether overexpression of YB-1 could confer oxaliplatin resistance in two colorectal cancer cell lines. Transient transfection experiment in SW480 and HT29 colorectal cancer cells and stable SW480 clones overexpressing a TAP-YB-1 construct all demonstrated a significant resistance to the drug oxaliplatin. From this information, we then asked whether a specific YB-1 protein complex is required to confer oxaliplatin resistance in SW480 and HT29 cells. We first identified by mass spectrometry analyses proteins binding directly to a TAP-YB-1 construct in SW480 cells. Sixty-seven proteins were identified with at least two peptides. To focus on proteins with the highest physiological relevance to chemioresistance in colorectal cancer, we opted for an integrative approach by collecting information on each protein with regard to gene expression and status (which includes genetic rearrangements) in colon cancers. Based on different public databases, 23 of these YB-1 partners were overexpressed at the transcriptional level in colorectal tumors, in metastasis, and associated with increased genomic instability in colon cancers. The transcription of one protein was decreased in recurring colon cancer. These observations indicated that the transcription levels of such proteins could potentially be used as prognostic markers. Based on microarray studies of different cell lines, the proteins HNRPL, PABPC1, RPS4X, RPS7, and RALY are overexpressed in ovarian cancer cells resistant to oxaliplatin [30]. From such information, we determined whether the depletion of these proteins up regulated in colorectal adenocarcinomas (Table 1) have an impact on oxaliplatin sensitivity on both SW480 and HT29 cancer cells. We used the knock down technology in this study as siRNA molecules can have potential therapeutic benefits. Importantly, the antisense technology has been described as a potential anti-cancer therapy for the treatment of several clinical cancers and several molecules are in phase I and II trials [23]. We tested four siRNA sequences for each target proteins in the list of Table 1. Different siRNA sequences for the same target often gave inconsistent results in the two cells lines used in this study (Table 2). Such results are not surprising as it is now well accepted that a siRNA sequence may present off-target effects that may differ depending on the transcriptome of each cell line [31-33]. We thus focused on proteins which showed at least two different siRNA sequences against the mRNAs corresponding to these proteins with the same phenotype in two different cancer cell lines. Based on these criteria, we observed that the depletion of RALY or NONO increased oxaliplatin sensitivity in both SW480 and HT29 cells. More importantly, the siRNA sequences showing increased oxaliplatin sensitivity efficiently depleted RALY or NONO proteins (Figure 6).

RALY (hnRNP-associated with lethal yellow homolog (mouse)) is a member of the heterogeneous nuclear ribonucleoprotein gene family. It is associated with the spliceosome complex [34]. It is unknown, however, whether RALY also affects transcription and DNA repair like YB-1 in cells [15,35,36]. One microarray study reported an increase of RALY expression in cells resistant to oxaliplatin along with the proteins HNRNPL, PABPC1, RPS4X, and RPS7 (Table 1) [30]. Our results, however, indicate that RALY is the only protein that sensitizes colorectal cancer cells to oxaliplatin when depleted with siRNAs. It is possible that HNRNPL, PABPC1, RPS4X, and RPS7 correlate with oxaliplatin resistance but are not directly involved biochemically in the process of resistance in colorectal cancer cell types. We recently found that a depletion of RPS4X increased cisplatin resistance in YB-1 overexpressing breast cancer cell lines [37]. We did not observe such cellular response in colorectal cancer cells upon oxaliplatin treatment in our study. This maybe due to the intrinsic difference between breast and colon cancer cell types, or it could be due to the different manner in which cells respond to cisplatin and oxaliplatin. For example, it has recently been reported that gamma irradiation of two teratoma cell lines *in vitro *induced resistance to subsequent oxaliplatin treatment, but increased sensitivity to cisplatin [38]. Notably, cisplatin and oxaliplatin DNA damage are not processed by the same DNA repair systems [39,40].

NONO (non-POU domain containing, octamer-binding) gene encodes an RNA-binding protein, which plays various roles in the nucleus, including transcriptional regulation and RNA processing [41-44]. A study with a GFP-NONO has indicated that the protein localizes not only to the nucleolus but also in nuclear speckles rich in splicing factors [41]. In addition to mRNA processing, NONO is involved in the repair of DNA double stranded breaks [45-47]. It is believed to participate in both the non-homologous end joining and the homologous recombination repair pathways with its homologue SFPQ (splicing factor proline/glutamine-rich). We did not identify, however, SFPQ in our mass spectrometry analyses as a protein interacting with the TAP-YB-1 construct.

Messenger RNA processing (which includes splicing) is a common biological process shared by YB-1, NONO, and RALY proteins [16,34,41]. Exon-array profiling is warranted in NONO, RALY, and YB-1 depleted cells to find common spliced mRNAs affected by these proteins during oxaliplatin response. Independent of the exact mechanisms involved in oxaliplatin resistance, NONO and RALY are good potential targets for the sensitization of otherwise oxaliplatin resistant YB-1 overexpressing colorectal cancer tumor cells (Figure 10). Interestingly, NONO or RALY knock down decreased endogenous YB-1 protein levels. The exact mechanism by which these proteins regulate YB-1 expression is unknown but our RT-PCR results indicate that a depletion of NONO also down regulates the level of YB-1 transcripts. In contrast, a depletion of RALY in SW480 cells did not significantly affect YB-1 mRNA levels. Thus, unlike NONO, RALY regulates the amount of YB-1 protein in SW480 cells at the post-transcriptional level. Additional experiments are required to determine which steps of YB-1 mRNA processing and transport NONO and RALY are involved in.

Finally, a depletion of YB-1 decreased NONO expression in SW480 cells but had no effect on RALY expression. This result is consistent with findings indicating that the promoter of NONO but not the promoter of RALY is a target of the YB-1 transcription factor [48]. It is possible that YB-1 regulates the transcription of the NONO gene in colorectal cancer cells. Epidemiological studies on appropriate cohorts of patients with colorectal cancer are warranted to determine whether the expressions of YB-1, NONO, and RALY have predictive values for the response to oxaliplatin treatment.

## Conclusions

Taken together, our results indicate that overexpression of YB-1 confers oxaliplatin resistance in colorectal SW480 and HT29 cancer cells. We show for the first time that YB-1 interacts with NONO and RALY in the colorectal cancer cell line SW480. Importantly, the knock down of either NONO or RALY sensitizes in otherwise oxaliplatin overexpressing YB-1 SW480 and HT29 colorectal cancer cell lines. These results suggest that knocking down NONO or RALY is a potential therapeutic strategy to counteract oxaliplatin resistance in colorectal cancers.

## Competing interests

The authors declare that they have no competing interests.

## Authors' contributions

SPT, HHY, and ML conceived and designed the experiments. SPT, CG, CS, DC, MA and DG performed the experiments. CG and DG generated the cells expressing the TAP-YB-1 constructs. SPT, CS, DC, MA HHY, and ML analyzed the data. ML and HHY wrote the paper. All authors read and approved the final manuscript.

## Supplementary Material

Additional file 1 **Table of the list of proteins identified by mass spectrometry in TAP-YB-1 eluate**. The total number of peptides identified by LC-MS/MS for each protein is indicated on the right. Click here for file

Additional file 2 **Oxaliplatin dose response curves and EC50 in SW480 and HT29 cells**. A) The graph on the left represents a twelve-point drug dose response (DDR) obtained with different control siRNAs in SW480 cells. The graph on the right represents the drug response curves obtained with the six concentrations used for the siRNA screening process in SW480 cells. B) The graph on the left represents a twelve-point drug dose response (DDR) obtained with different control siRNAs in HT29 cells. The graph on the right represents the drug response curves obtained with the six concentrations used for the siRNA screening process in HT29 cells. No tfx indicates no transfection. Mock tfx indicates transfection without a siRNA molecule. NS and All-Star NS (ASNS) are nonspecific siRNA sequences. GFP indicates siRNA against Green Fluorescent Protein. ACDC stands for Allstar Cell death Control. EC50 values were calculated from the oxaliplatin dose response curves obtained after transfection of the indicated siRNA sequences. Experiments were performed in duplicate. Click here for file

Additional file 3 **Impact of siRNAs on SW480 colorectal cancer cell viability**. The tables in this file present the raw data on the viability of SW480 cells after the transfections of siRNA sequences. Click here for file

Additional file 4 **Impact of depleting NONO and RALY proteins on YB-1 levels in SW480 cells**. Examples of Western blots showing YB-1, NONO, and RALY expression four days after the transfections with the siRNA sequences A) siNONOa and siNONOb and B) siRALYa and siRALYc molecules. Click here for file

Additional file 5 **Microarray data showing high expression of YBX1, NONO, or RALY mRNAs in different sets of cancer patients with poor outcome of the disease**. The table gives a list of microarray data set showing an association between poor outcomes of cancer patients with high expression of YB-1, NONO, and/or RALY transcripts. The cancer type, the probe for each transcript, and the array type used in the studies are provided with P-values for the association. Highlighted are studies showing co-expression of the three different genes. Click here for file
