# Supplementary material for: NONO and RALY proteins are required for YB-1 oxaliplatin induced resistance in colon adenocarcinoma cell lines
Source: Mol Cancer. 2011 Nov 25;10:145. doi: 10.1186/1476-4598-10-145 (PMC3240900; doi:10.1186/1476-4598-10-145)
Supplement: Additional file 2 — Oxaliplatin dose response curves and EC50 in SW480 and HT29 cells. A) The graph on the left represents a twelve-point drug dose response (DDR) obtained with different control siRNAs in SW480 cells. The graph on the right represents the drug response curves obtained with the six concentrations used for the siRNA screening process in SW480 cells. B) The graph on the left represents a twelve-point drug dose response (DDR) obtained with different control siRNAs in HT29 cells. The graph on the right represents the drug response curves obtained with the six concentrations used for the siRNA screening process in HT29 cells. No tfx indicates no transfection. Mock tfx indicates transfection without a siRNA molecule. NS and All-Star NS (ASNS) are nonspecific siRNA sequences. GFP indicates siRNA against Green Fluorescent Protein. ACDC stands for Allstar Cell death Control. EC50 values were calculated from the oxaliplatin dose response curves obtained after transfection of the indicated siRNA sequences. Experiments were performed in duplicate. [file 1476-4598-10-145-S2.PDF]

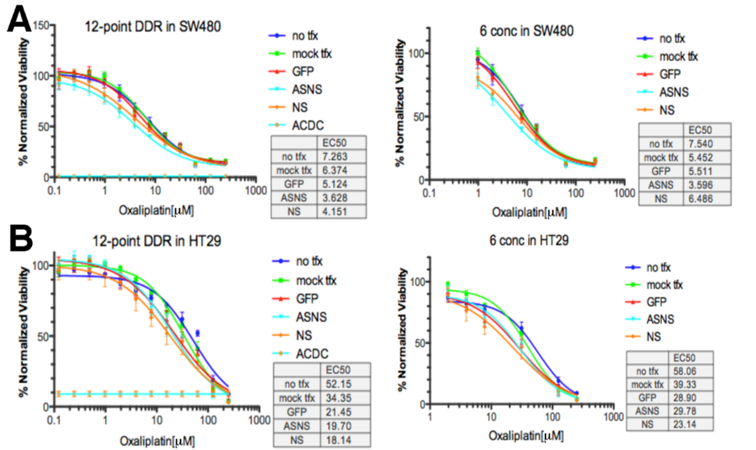

Additional File 2. *Oxaliplatin dose response curves and EC50 in SW480 and HT29 cells*. A) The graph on the left represents a twelve-point drug dose response (DDR) obtained with different control siRNAs in SW480 cells. The graph on the right represents the drug response curves obtained with the six concentrations used for the siRNA screening process in SW480 cells. B) The graph on the left represents a twelve-point drug dose response (DDR) obtained with different control siRNAs in HT29 cells. The graph on the right represents the drug response curves obtained with the six concentrations used for the siRNA screening process in HT29 cells. No tfx indicates no transfection. Mock tfx indicates transfection without a siRNA molecule. NS and All-Star NS (ASNS) are nonspecific siRNA sequences. GFP indicates siRNA against Green Fluorescent Protein. ACDC stands for Allstar Cell death Control. EC50 values were calculated from the oxaliplatin dose response curves obtained after transfection of the indicated siRNA sequences. Experiments were performed in duplicate.
