# Supplementary material for: NONO and RALY proteins are required for YB-1 oxaliplatin induced resistance in colon adenocarcinoma cell lines
Source: Mol Cancer. 2011 Nov 25;10:145. doi: 10.1186/1476-4598-10-145 (PMC3240900; doi:10.1186/1476-4598-10-145)
Supplement: Additional file 4 — Impact of depleting NONO and RALY proteins on YB-1 levels in SW480 cells. Examples of Western blots showing YB-1, NONO, and RALY expression four days after the transfections with the siRNA sequences A) siNONOa and siNONOb and B) siRALYa and siRALYc molecules. [file 1476-4598-10-145-S4.PDF]

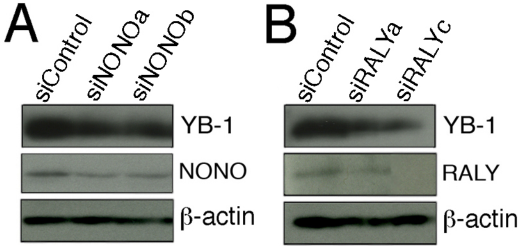

Additional File 4. *Impact of depleting NONO and RALY proteins on YB-1 levels in SW480 cells*. Examples of Western blots showing YB-1, NONO, and RALY expression four days after the transfections with the siRNA sequences A) siNONOa and siNONOb and B) siRALYa and siRALYc molecules.
